# Virulence adaptation in a rice leafhopper: Exposure to ineffective genes compromises pyramided resistance

**DOI:** 10.1016/j.cropro.2018.07.010

**Published:** 2018-11

**Authors:** Finbarr G. Horgan, Carmencita C. Bernal, Quynh Vu, Maria Liberty P. Almazan, Angelee Fame Ramal, Hideshi Yasui, Daisuke Fujita

**Affiliations:** a University of Technology Sydney, 15 Broadway, Ultimo, Sydney, NSW, 2007, Australia; b Tropical Ecosystems Research Network, 30C Nirondha, Temple Road, Piliyandala, Sri Lanka; c International Rice Research Institute, DAPO Box, 7777, Metro Manila, Philippines; d Cuulong Delta Rice Research Institute, Tan Thanh, Thoi Lai District, Can Tho, Viet Nam; e Helmholtz Centre for Environmental Research - UFZ, Theodor-Lieser-Str. 4, 06120, Halle, Germany; f School of Environmental Science and Management, University of the Philippines, Los Baños, 4030, Laguna, Philippines; g Plant Breeding Laboratory, Graduate School, Kyushu University, Fukuoka, 812-8581, Japan; h Saga University, Faculty of Agriculture, 1 Honjo-machi, Saga, 840-8502, Japan

**Keywords:** Brown planthopper, Crop improvement, Integrated pest management, Marker-assisted breeding, Philippines, Resistance durability, Resistance management

## Abstract

Pyramiding resistance genes is predicted to increase the durability of resistant rice varieties against phloem-feeding herbivores. We examined responses by the green leafhopper, *Nephotettix virescens* (Hemiptera: Cicadellidae), to near-isogenic rice lines with zero, one and two resistance genes. The recurrent parent (T65) and monogenic lines (*GRH2-*NIL and *GRH4-*NIL) with genes for resistance to the green rice leafhopper, *Nephotettix cincticeps* (Hemiptera: Cicadellidae), were susceptible to the green leafhopper, but the pyramided line (*GRH2/GRH4-*PYL) was highly resistant to the green leafhopper. We selected green leafhoppers, *N. virescens*, from five sites in the Philippines for over 20 generations on each of the four lines. Populations selected on *GRH2/GRH4-*PYL gained partial virulence (feeding and development equal to that on T65) to the pyramided line within 10 generations and complete virulence (egg-laying equal to that on T65) within 20 generations. After 20 generations of rearing on the susceptible monogenic lines, green leafhoppers were also capable of developing and laying eggs on *GRH2/GRH4-*PYL. Furthermore, green leafhoppers reared on the susceptible *GRH4-*NIL for 20 generations showed equal preferences for T65 and *GRH2/GRH4-*PYL in choice bioassays. Our results indicate that previous long-term exposure to ineffective genes (including unperceived resistance genes) could dramatically reduce the durability of pyramided resistance. We suggest that informed crop management and deployment strategies should be developed to accompany rice lines with pyramided resistance and avoid the build-up of virulent herbivore populations.

## Highlights

• Monogenic rice lines with *GRH2* and *GRH4* loci were susceptible to green leafhopper.
• A pyramided line with both genes was highly resistant to the leafhopper.
• Populations selected for 20 generations adapted to feed and oviposit on the pyramided line.

## 1 Introduction

Crop improvement increasingly relies on advanced molecular techniques to accelerate breeding pipelines by targeting specific traits of interest and avoiding undesirable trade-offs (Varshney et al., 2005; Wang et al., 2005; Collard and Mackill, 2008). A major objective of rice improvement has been to use marker-assisted selection (MAS) to increase resistance against a range of insect herbivores. In recent years, a number of research papers have described new anti-herbivore resistance genes/loci, identified useful genetic markers to support breeding programs, or developed advanced breeding lines with enhanced resistance to insect herbivores (Fujita et al., 2013; Bentur et al., 2016; Hu et al., 2016). Rice leafhoppers (Hemiptera: Cicadellidae) and planthoppers (Hemiptera: Delphacidae) are among the principal targets of molecular breeding for resistance in rice (Fujita et al., 2013; Horgan, 2018). Leafhoppers occur throughout tropical rice growing regions. They occasionally cause mechanical damage to rice plants, including ‘hopperburn’ (the drying and wilting of rice plants in large patches) and are vectors of rice diseases (i.e., tungro viruses transmitted by *Nephotettix virescens* [Distant] and *Recilia dorsalis* Motschulsky, and rice dwarf disease and transitory yellowing disease transmitted by *Nephotettix cincticeps* [Uhler]) (Azzam and Chancellor, 2002; Asano et al., 2015).

A range of genes for resistance to rice leafhoppers has been identified (Fujita et al., 2013). However, leafhopper virulence adaptation (the selection of populations able to feed and develop on resistant hosts) is often rapid and many genes are only locally effective (Sato and Sogawa, 1981; Heinrichs and Rapusas, 1985; Dahal et al., 1997). Virulence adaptation can be partial or complete (Heinrichs and Rapusas, 1985; Dahal et al., 1997; Vu et al., 2014). Partial adaptation in leafhoppers can occur in as little as 5–6 generations of selection (Vu et al., 2014). Pyramiding resistance genes (i.e., combining two or more genes in a single line) has been proposed to reduce rates of virulence adaptation in insect herbivores (Wang et al., 2016; Fan et al., 2017). Several authors have described protocols associated with pyramiding resistance genes in rice and verified that the resulting resistance is stronger than that from monogenic rice lines (Fujita et al., 2010; Wang et al., 2016; Fan et al., 2017). However, there is still little information to assess pyramiding as a strategy to prolong field resistance (Horgan, 2018). Indeed, without informed deployment strategies, pyramiding resistance could result in more rapid losses of resistance genes than if the genes had been sequentially deployed in monogenic lines (Cheng, 1985; Nemoto and Yokoo, 1994; Horgan, 2018).

Zhao et al. (2005) used a series of replicated mesocosms to examine adaptation by the diamondback moth, *Plutella xylostella* (Linneaus), to pyramided Cry1Ac and Cry1C genes in *Bt*-transgenic broccoli. Their results indicated that the concurrent deployment of *Bt* genes in monogenic and pyramided lines can dramatically reduce the durability of pyramided resistance. In mesocosms with monogenic and pyramided resistant lines, the moth populations first overcame the monogenic resistance (either Cry1Ac or Cry1C) before sequentially adapting to the remaining effective gene in the pyramided line. In this example of *Bt* transgenic broccoli, moth populations adapted to detoxify two independently functioning toxins. However, the potential effects of concurrently deploying conventionally bred monogenic and pyramided resistance crops are more difficult to predict. This is because conventional resistance can depend on networks of interacting genes (Fujita et al., 2013), with the same resistance genes often producing different effects depending on the genetic background of the host plant (Heinrichs and Rapusas, 1985; Cohen et al., 1997; Alam and Cohen, 1998; Peñalver Cruz et al., 2011). Furthermore, pyramiding resistance loci in rice can result in strong resistance even where each of the loci are ineffective in monogenic lines (e.g., *GRH2* and *GRH4*: Vu et al., 2014; *BPH25* and *BPH26*: Srinivasan et al., 2015). Deploying susceptible varieties that possess ineffective and potentially unperceived resistance genes could therefore threaten the utility of pyramiding certain gene combinations.

In the present study, we use a series of near-isogenic rice lines with zero, one and two genes/loci for resistance against leafhoppers. The lines were developed by MAS using donor varieties with known resistance against the green rice leafhopper, *N. cincticeps*. Although the improved lines (with one or two resistance loci) can be moderately or strongly resistant to *N. cincticeps* (Fujita et al., 2010), only the pyramided line is resistant to the closely related green leafhopper, *N. virescens* (Vu et al., 2014). This herbivore-plant system provided us with an opportunity to examine aspects of virulence adaptation to pyramided lines and to test whether monogenic plants with ineffective genes could accelerate adaptation by leafhoppers to pyramided lines with the same genes. We therefore (a) describe virulence adaptation to the pyramided line using replicated green leafhopper colonies, and (b) determine whether exposure to monogenic lines predisposes populations to virulence against pyramided lines with the same resistance gene(s). We discuss our results in the light of sustainable deployment of pyramided lines to increase the durability of field resistance and to preserve rare resistance genes.

## 2 Materials and methods

### 2.1 Plant materials

We used monogenic near-isogenic-lines (NILs) carrying either the *GRH2* or *GRH4* gene loci (henceforth *GRH2-*NIL and *GRH4-*NIL, respectively) and a pyramided line carrying both genes together (henceforth *GRH2/GRH4-*PYL [we use ‘PYL’ to indicate a near-isogenic line with pyramided, ≥ 2 resistance genes]) in our experiments with *N. virescens*. The monogenic and pyramided near-isogenic lines were originally developed using marker assisted selection. The resistance genes on either locus (*GRH2* or *GRH4*) have not yet been cloned.

The two genes, *GRH2* and *GRH4*, were first identified from DV85 using the green rice leafhopper, *N. cincticeps* during plant phenotyping (Fujita et al., 2010, 2013). Previous studies demonstrated that *GRH2* in monogenic NILs produced resistance against *N. cincticeps*, but that the PYL (containing both genes) developed using T65 as a recurrent parent, had notably higher resistance (Fujita et al., 2013; Asano et al., 2015).

DV85 and T65 were obtained from the Germplasm Bank at the International Rice Research Institute (IRRI) in the Philippines. The resistant lines we used were BC_6_F_5_ generations selected using Simple Sequence Repeat markers associated with the target loci during repeated backcrossing of the donor variety DV85 and the recurrent parent T65 (Fujita et al., 2010). Seed of the NILs was bulked-up in a screenhouse at IRRI during the dry-season when temperatures were coolest.

In all experiments, rice plants were grown in size-zero terracotta pots (5 × 2.5 cm: Height × Radius [H × R]) filled with paddy soil. The pots were held in flooded metal trays to maintain soil at saturation.

### 2.2 Green leafhopper colonies

In this study, we used a range of greenhouse colonies derived from five initial *N. virescens* populations (Fig. S1). One colony (henceforth, ‘Los Baños’) was initiated in 2008 using wild-caught individuals from Los Baños, Laguna Province in Southern Luzon, the Philippines. Four further colonies were initiated in 2010 using *N. virescens* collected at four locations (Batangas, Quezon, Rizal and San Pablo - Laguna) in Southern Luzon. These sites were each separated by distances of between 10 and 30 Km (See Fig. S1).

Colonies were initiated with ca. 500 adults collected from rice fields at the sites and placed on the susceptible rice variety TN1 (≥30-day old rice plants) in wire mesh cages of 120 × 60 × 60 cm, Height × Width × Length [H × W × L]) under greenhouse conditions (temperatures ranged from 25 to 37 °C, 12D:12N photoperiod). During the first two generations of rearing, the colonies were synchronized (such that key life-stages were available at any one time across all five colonies). During these initial generations, the colonies were also monitored for possible transmission of rice viruses. Based on the health of feeding plants (these did not yellow or show other symptoms of virus), we assumed that the leafhoppers did not transmit tungro virus.

After two or three generations, a series of bioassays was conducted to determine population reactions to the test rice lines. The methods and results of these bioassays are presented in the supplementary information (Tables S1 and S2). Bioassays indicated that each of the populations was largely virulent against rice lines with the *GRH2* or *GRH4* gene loci (monogenic), but that all populations were avirulent against rice lines with both genes together. The Los Baños and Rizal populations had delayed nymph development on a rice line with the *GRH4* gene compared to the other three populations, but nymph survival and weight gain on this line were not significantly different from the other populations (full results are available in Table S2).

### 2.3 Multi-generation selection and monitoring of leafhopper colonies

After two or three generations on TN1, the five populations were each divided into four parts and placed in separate cages with either T65, *GRH2*-NIL, or *GRH4*-NIL (ca 200 adult pairs) or with *GRH2/GRH4-*-PYL (ca 500 pairs). The larger number of pairs on the pyramided line was to overcome initial high mortality of adults and low rates of oviposition (Table S2). For the purposes of this paper, the rice lines on which colonies were selected are referred to as ‘natal hosts’. Plants used in monitoring and other bioassays are ‘exposed hosts’. Nymph and adult survival were monitored at generations 1, 2, 3, 5, 7, 10, and 20–26. Egg laying was assessed at generations 10, and 20–26. Details of the bioassays are presented in Table S1 and explained briefly here.

To assess adult and nymph survival, newly emerged nymphs and gravid females (isolated as fifth instars to ensure they were unmated), respectively, were collected from each colony (ten neonates or five adults per plant) and placed on plants of the same rice lines on which they were being selected (exposed hosts = natal hosts) at 20 days after sowing (DAS). The plants were grown in size-0 pots under acetate insect cages (45 × 2.5 cm: H × R). The cages fitted neatly over the plants and into the pots. Each cage had a side-window and top of insect-proof netting. After 15 days, the number of survivors on each plant and their development stages were recorded. The survivors were then collected and dried in a forced draught oven at 60 °C for 3 days before being weighed. The host plants were also dried and weighed. Egg-laying was monitored by introducing mated, gravid females (two females) to 20 DAS plants grown in size-0 pots under insect cages (dimensions as above). The females were allowed to oviposit for 5 days after which the plants were collected and dissected to count the eggs. The plants were then dried and weighed (described above).

To ensure that stable virulence was achieved after 20 generations, green leafhoppers from the *GRH2/GRH4-*PYL selected colonies were placed on T65 for six generations and evaluated for their fitness on and preferences for the pyramided line. Results of these bioassays are presented in Figs. S2 and S3.

### 2.4 Propensity for selected populations to develop on *GRH2/GRH4-*PYL

The continuous rearing of green leafhoppers on *GRH2*-NIL and *GRH4*-NIL for 20 generations allowed us to test whether exposure to ineffective resistance genes predisposed the leafhoppers to virulence against the pyramided resistant line.

Leafhoppers from each of the colonies (5 origins × 4 natal hosts = 20 colonies) were examined for their ability to survive and develop on *GRH2/GRH4*-PYL (= exposed host). Nymph survival, adult survival and oviposition no-choice bioassays (described above and see Table S1) were conducted.

A series of choice bioassays were also conducted with each of the selected colonies (5 populations × 4 natal hosts = 20 colonies per bioassay) after 20 generations to examine their preferences between the recurrent parent T65 and the *GRH2/GRH4-*PYL (Table S2). Bioassays were conducted as described above (section 2.3).

### 2.5 Data analyses

Changes in the fitness of leafhoppers during selection were analysed using repeated measures general linear models (GLM). Because three of the four lines were largely ineffective in reducing leafhopper fitness, and fitness on the remaining line improved over generations, we expected significant generation (repeated measure) by rice line interactions for all fitness parameters. Changes in fitness over the generations of selection are presented graphically as deviations from fitness observed with corresponding colonies (i.e., from the same geographical origin) on the recurrent parent (T65).

Non-choice bioassays were analysed using GLM. The results of binary choice experiments were expressed as the proportions of leafhoppers showing preferences for either T65 or *GRH2/GRH4-*PYL. Average proportions approaching 0.5 indicated no preference and, therefore, adaptation for settling or egg-laying on the resistant line. Average preferences for *GRH2/GRH4-*PYLs among colonies reared on the four natal hosts were compared using univariate GLMs.

Colony origin was included in all models as a blocking factor. Plant biomass was initially included in all models as a covariate and was later removed where it had no effect. Post-hoc Tukey tests were conducted for the factor ‘natal host’ after each analysis. Residuals were plotted after all parametric analyses and found to be normal and homogeneous. Analyses were conducted using SPSS v.22 (IBM SPSS, Armonk, NY, USA).

## 3 Results

### 3.1 Green leafhopper fitness on natal hosts during selection

**Fig. 1:**
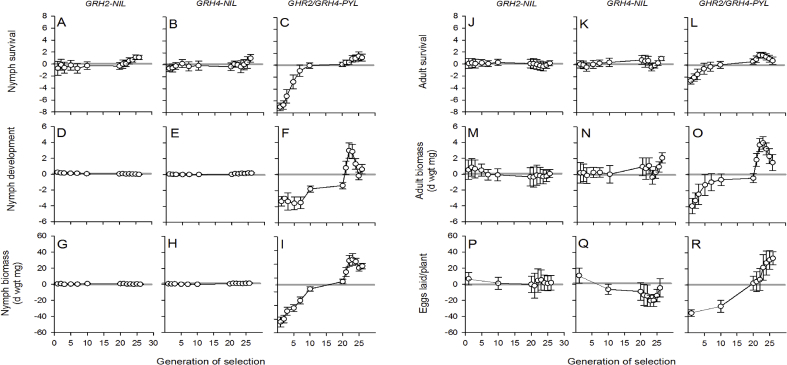
Nymph survival (A–C), nymph development (the proportion reaching adult stage) (D–F) and nymph biomass (G–I) from nymph survival bioassays, with adult survival (J–L) and adult biomass (M–O) from adult survival bioassays, and eggs-laid (P–R) in oviposition bioassays relative to values on T65. Details of bioassays are presented in Table S1. Bioassays were conducted with leafhoppers from colonies selected over 26 generations on *GRH2-*NIL (A,D,G,J,M,P), *GRH4-*NIL (B,E,H,K,N,Q) and *GRH2/GRH4-*PYL (C,F,I,L,O,R) with results presented relative to green leafhopper colonies reared on T65 (indicated by the grey lines at 0 in each figure). Relative measures were calculated as (fitness on the natal host (with resistance gene/s) – fitness on recurrent parent T65). Fitness bioassays for each natal host were conducted simultaneously within each generation. Error bars are standard errors of means (N = 5 colonies).

Wild green leafhopper populations collected from five sites in southern Luzon were capable of feeding, surviving and laying eggs on *GRH2*-NIL and *GRH4*-NIL, but had significantly lower survival and egg-laying on *GRH2/GRH4*-PYL (Table S2, Fig. 1). When confined in cages (colonies) with *GRH2/GRH4*-PYL (as the natal host), the colonies adapted within 5–10 generations to achieve high survival and weight gains (equivalent to colonies on T65) when feeding on the pyramided line (Fig. 1 C,F). However, the numbers of eggs laid on the *GRH2/GRH4*-PYL remained lower than on T65 even at the tenth generation (Fig. 1 I).

When monitored during the 20th generation, all colonies had adapted to lay-eggs on *GRH2/GRH4*-PYL (ovipositing as many eggs as on T65: Fig. 1 I). Improved fitness of all colonies on the *GRH2/GRH4*-PYL natal host through selection resulted in significant generation effects (including significant linear contrasts for generation) and significant generation × natal host interactions (Table 1).

**Table 1:** Results from repeated measure GLM for leafhopper fitness parameters over 26 generations of selection (sampled at generations 1, 2, 3, 5, 7, 10, and 20–26; oviposition was sampled only at generations 10 and 20-26).

Sources of variation	DF^a^	F-values^b^					
		Adult survival	Adult biomass	Number of eggs laid	Nymph survival	Nymph biomass	Nymph development
*Within subject effects*							
Generation	12 (8)	8.923***	7.457***	3.969***	12.519***	16.634***	9.304***
Linear contrast	1	4.559*	0.001	5.245*	63.949***	96.860***	5.023*
Generation × natal host	36 (24)	2.490***	5.598***	4.259***	3.447***	7.086***	3.250***
Error	192 (128)						
*Between subject effects*							
Natal host^c^	3	1.407	3.346*	3.013	5.679**	19.013***	1.192
Error	16						

a Numbers in parentheses are degrees of freedom for oviposition bioassay.

b *** = P < 0.001, ** = P < 0.01, * = P < 0.05.

c In all significant cases, *GRH2/GRH4-*PYL is different from all other lines.

During further rearing on *GRH2/GRH4*-PYL (generations 21–26), the selected colonies often performed significantly better on the PYL than on equivalent colonies continuously reared on T65 (Fig. 1).

### 3.2 Propensity for selected populations to develop on *GRH2/GRH4-*PYL

**Fig. 2:**
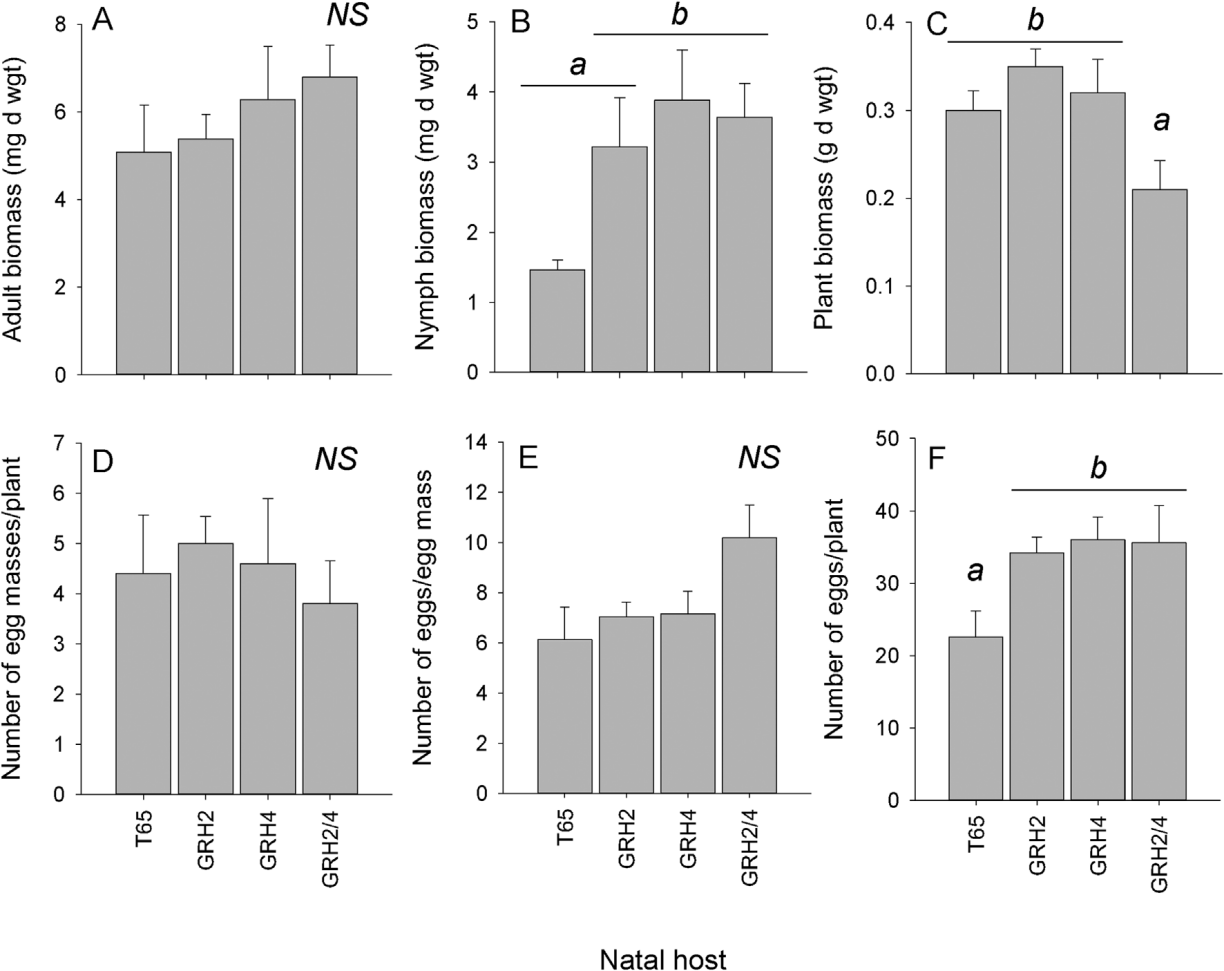
Results of fitness bioassays with colonies reared for 20 generations on T65, *GRH2-*NIL, *GRH4-*NIL or *GRH2/GRH4-*PYL and exposed to *GRH2/GRH4-*PYL. Graphs indicate (A) the biomass of adults after 15 days, (B) the biomass of nymphs after 15 days, (C) the final biomass of the host *GRH2/GRH4-*PYL plants after 15 days of nymph feeding, (D) the number of egg masses per plant, (E) the average size of egg masses and (F) the number of eggs per plant. Lowercase letters indicate homogenous groups (Tukey, P ≤ 0.05); standard errors are indicated (N = 5 colonies).

**Fig. 3:**
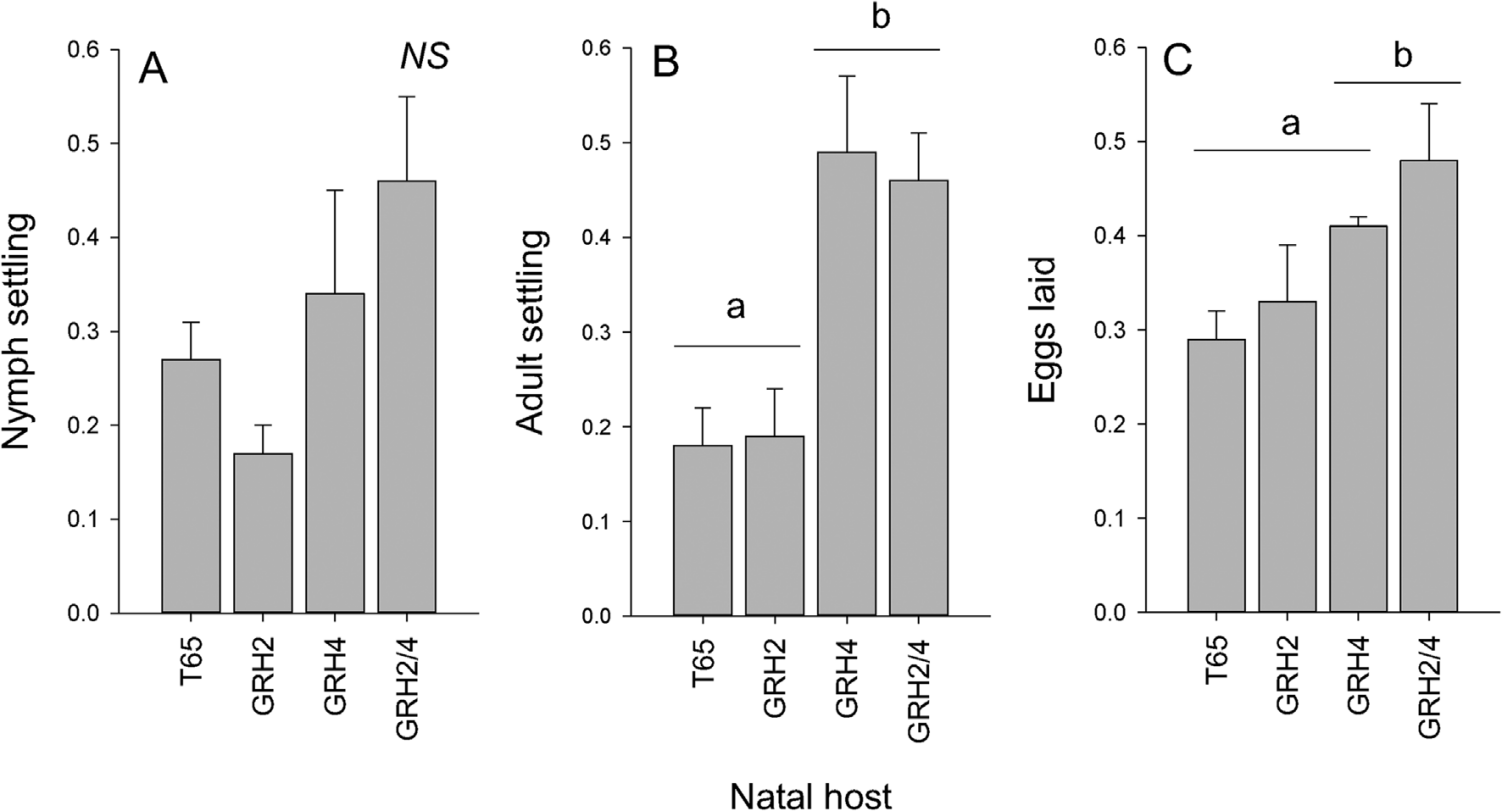
Proportion of nymphs (A) and adults (B) settling on *GRH2/GRH4-*PYL in choice bioassays, with (C) eggs laid on *GRH2/GRH4-*PYL as a proportion of the total number of eggs laid in the bioassays (i.e., eggs on PYL/(eggs on PYL + eggs on T65)). Bioassays were conducted with colonies selected on T65, *GRH2-*NIL, *GRH4-*NIL or *GRH2/GRH4-*PYL (indicated as ‘T65″, ‘GRH2″, ‘GRH4’ and ‘GRH2/4’, respectively on the x-axes) for 20 generations. Lowercase letters indicate homogenous groups (Tukey tests, P ≤ 0.05); standard errors are indicated (N = 5 colonies).

Leafhoppers that were continuously maintained on *GRH2-*NIL and *GRH4*-NIL had significantly improved their ability to survive and lay eggs on the *GRH2/GRH4*-PYL (Fig. 2, Fig. 3).

Adult (76–84%) and nymph survival (100%) on *GRH2/GRH4-*PYL during the no-choice bioassays was high and was not affected by natal host. Adult biomass was not affected by natal host (F_3,16_ = 0.723, P = 0.553: Fig. 2 A). The biomass of green leafhopper nymphs selected on the monogenic NILs and exposed to *GRH2/GRH4-*PYL was not different from green leafhopper nymphs continuously reared on the pyramided line (F_3,16_ = 3.878, P = 0.029: Fig. 2 B). Despite the similar survival and weight gain of leafhoppers selected on the monogenic and pyramided lines, the final biomass of the exposed plants (*GRH2/GRH4-*PYL) was lower when attacked by the *GRH2/GRH4-*PYL-selected leafhoppers (F_3,16_ = 4.098, P = 0.025: Fig. 2 C).

Green leafhoppers reared on the different natal hosts produced similar numbers of egg masses on *GRH2/GRH4-*PYL (F_3,16_ = 0.246, P = 0.863: Fig. 2 D); the masses produced by the *GRH2/GRH4-*PYL-selected leafhoppers were generally, but not statistically significantly larger than those produced by the other colonies (F_3,16_ = 2.752, P = 0.077: Fig. 2 E). However, overall more eggs were laid on *GRH2/GRH4-*PYL by green leafhoppers that were selected on the monogenic and pyramided lines than on T65 (F_3,16_ = 3.469, P = 0.041: Fig. 2 F).

When presented with both T65 and *GRH2/GRH4*-PYL in choice bioassays, green leafhoppers that were exposed for 20 generations to *GRH4*-NIL showed no differences in their preferences (nymph settling, adult settling and egg-laying) for either T65 or *GRH2/GRH4-*PYL (nymph settling F_3,19_ = 2.159, P > 0.05 = no difference among colonies from all natal hosts: Fig. 3 A; adult settling F_3,19_ = 7.622, P ≤ 0.01 = no difference between *GRH4-*NIL and *GRH2/GRH4-*PYL as natal hosts: Fig. 3 B; egg-laying F_3,19_ = 3.457, P ≤ 0.05 = no difference between *GRH4-*NIL and *GRH2/GRH4-*PYL as natal hosts: Fig. 3 C).

## 4 Discussion

### 4.1 Pyramiding and resistance strength

Despite a lack of any obvious effects of the *GRH2* and *GRH4* loci on leafhopper population development, we found that the pyramided line *GRH2/GRH4*-PYL was highly resistant to green leafhoppers. In a related study, Vu et al. (2014) indicated that the resistance of *GRH2/GRH4-*PYL to green leafhoppers is stable throughout plant development and under high soil nitrogen. Vu et al. (2014) also indicated that *GRH4-*NIL infested at 45 DAS, was moderately resistant to green leafhoppers, but plants attacked at other stages (10, 30 and 60 DAS) were as susceptible to leafhoppers as T65. In the present study, we also noted that among the two monogenic NILs, *GRH4*-NIL had indications of stronger effects on green leafhoppers than *GRH2*-NIL: the original Los Baños, Batangas and Rizal field populations had notably slower nymph development on *GRH4*-NIL than on *GRH2*-NIL or T65, but no other effects were observed and damage to the monogenic NILs was severe. This differs from the responses by *N. cincticeps* where the *GRH2* gene is associated with low nymph survival, but the *GRH4* gene had little effect on nymphs (Fujita et al., 2010; Asano et al., 2015). Nevertheless, pyramiding these genes increases the strength of resistance against *N. cincticeps* (Fujita et al., 2010; Asano et al., 2015). Similar cases of combining one or two largely ineffective genes in pyramided lines to produce strong resistance have been noted previously. For example, the *BPH25* and *BPH26* genes against Philippine populations of the brown planthopper, *Nilaparvata lugens* (Stål) are ineffective in monogenic lines, but when pyramided they bestow strong resistance to the planthopper (Srinivasan et al., 2015). In both these cases, the resistance genes were originally derived from a single rice variety, (i.e., *GRH2* and *GRH4* from DV85, and *BPH25* and *BPH26* from ADR52). It is therefore unsurprising that when the genes were separated through backcrossing of the resistance donors and the recurrent parent T65 (in both cases), that the monogenic near isogenic lines would result in weaker resistance. However, in a study with *Bph1* (from Mudgo) and *bph2* (from ASD7), a pyramided line was resistant to planthoppers that were virulent against both genes in monogenic lines (Seo et al., 2010). Pyramiding genes can therefore result in novel resistance from otherwise ineffective genes.

### 4.2 Mechanisms of resistance

Previous studies with *N. cincticeps* on *GRH2* and *GRH4* did not examine effects of the individual genes or the pyramided line on egg laying (Fujita et al., 2010; Asano et al., 2015). The present study indicates that a pyramided line reduced feeding efficiency (as determined from honeydew excretion bioassays), survival and egg-laying in *N. virescens*. Although reduced egg-laying could be related to the inability of adult leafhoppers to feed on the host plant, partial adaptation by the colonies to feed but not lay eggs on the resistant line, suggests that feeding and egg-laying are affected by different components of resistance.

Using microarray analysis, Asano et al. (2015) linked the *GRH2* gene to a defence response against *N. cincticeps*, but suggested that plants with both *GRH2* and *GRH4* had more effective defence responses. Asano et al. (2015) indicated that infestation by *N. cincticeps* of a *GRH2/GRH4*-PYL (TGRH29) resulted in the expression of genes for several types of proteinase inhibitor and several genes from the cytochrome P450 family. These genes were not expressed after equivalent *N. cincticeps* attacks on T65. These authors also indicated that TPS genes were highly upregulated in response to attacks by *N. cincticeps* on the *GRH2/GRH4-*PYL. These included genes associated with the production of volatiles. In particular, Asano et al. (2015) indicated a strong induction of sesquiterpenes (zingiberene, β-sesquiphellandrene and β-bisabolene) in the *GRH2/GRH4-*PYL following *N. cincticeps* attack. Although these studies were conducted with *N. cincticeps*, it is probable that similar responses occur when plants are attacked by the closely related green leafhopper, *N. virescens*.

In our study, *N. virescens* ingested higher amounts of xylem sap when feeding on *GRH2/GRH4*-PYL indicating that the leafhoppers were either incapable of locating the phloem tubes or that they attempted to detoxify or dilute defence chemicals using xylem fluids (Ferrater et al., 2015). Inefficient feeding, as observed in this study, could result from volatile emissions in response to attack and/or from the anti-digestion effects of proteinase inhibitors. The mechanisms underlying reduced oviposition on *GRH2/GRH4-*PYL have not been investigated, but might also relate to volatiles emitted during insect attack. For example, in our no-choice bioassays, leafhoppers without previous exposure to *GRH2/GRH4-*PYL laid generally more, but smaller egg masses than leafhoppers selected on the pyramided line. In a related study (Horgan et al., unpublished), we found no evidence of an induced ovicidal response in the pyramided line.

### 4.3 Pyramiding genes for durable resistance

Pyramiding resistance genes against phloem-feeding herbivores is largely a response to the rapid adaptation by planthoppers and leafhoppers to resistant rice varieties (Bottrell and Schoenly, 2012; Horgan and Crisol, 2013; Horgan, 2018). The first monogenic resistant varieties released in Asia during the 1970s and 1980s were overcome by planthoppers in as little as 2 years (12 generations), but several traditional varieties with polygenic resistance have continued to be highly resistant to leafhoppers and planthoppers in screening studies (e.g., PTB33, Rathu Heenati: Horgan et al., 2015, 2017). In our study, leafhoppers adapted to feed on *GRH2/GRH4-*PYL within 5–10 generations and to oviposit on *GRH2/GRH4-*PYL within 20 generations. Furthermore, when these adapted colonies were returned to T65 for six generations, they did not lose their virulence against the pyramided line. Previous selection studies, have indicated that leafhoppers can quickly lose virulence when returned to susceptible varieties (e.g., 2 generations: Takita and Habibuddin, 1985; 7 generations: Dahal et al., 1997). However, because these studies did not examine adaptation for egg laying, it is unknown whether the populations were partially or completely adapted to the respective natal hosts. In a study by Heinrichs and Rapusas (1985) that clearly indicated leafhopper adaptation to lay eggs on resistant rice, virulence was not reversed after the leafhoppers had been returned to susceptible varieties. In similar studies with the brown planthopper, virulence was also not reversed when colonies were returned to susceptible hosts (Claridge and Den Hollander, 1982; Myint et al., 2009a, 2009b).

Several studies have recently reported increased resistance strength and high yields in field exposures of rice lines with pyramided resistance against planthoppers (Li et al., 2011; Hu et al., 2013; Qiu et al., 2012; Liu et al., 2016; Wang et al., 2017; Jiang et al., 2018). However, none of these studies has investigated rates of adaptation to pyramided resistance or examined whether pyramiding prolongs resistance beyond that expected from the sequential deployment of equivalent genes in monogenic lines. In a study with green rice leafhoppers, Hirae et al. (2007) found a *N. cincticeps* population to improve feeding and egg laying on *GRH2* (Saikai 182) within 8 generations of selection. However, in the same study, leafhoppers failed to adapt to Norin PL5 (with both *GRH2* and *GHR4*). Green rice leafhoppers reared on the line with both *GRH2* and *GRH4* had such high mortality that selected populations could not be maintained beyond three generations. Pyramiding may function to increase resistance durability by producing sufficiently strong resistance to avoid the establishment of focal herbivore populations. However, it is increasingly apparent that individuals (called ‘forerunners’ by Ketipearachchi et al. (1998)) that are unaffected by certain resistance genes occur in small numbers in wild planthopper and leafhopper populations. These individuals may possess virulence genes that are closely associated with specific resistance genes (i.e., Jing et al., 2014; Kobayashi et al., 2014). Because virulence against ≥2 genes is common among planthoppers and leafhoppers (Myint et al., 2009a,2009b; Horgan et al., 2015, 2017), forerunners with several virulence genes may also exist, although at lower frequencies (Horgan, 2018). Furthermore, planthoppers (and possibly leafhoppers) that overcome one gene, will often gain virulence to other, unrelated genes (e.g., *BPH8* and *BPH9*: Ketipearachchi et al., 1998; *BPH3/BPH32* and *BPH4*: Peñalver Cruz et al., 2011). Overall, and despite the problems and costs associated with virulence adaptation, little is known about the adaptation mechanisms of planthoppers and leafhoppers to resistant rice.

Our original wild populations were largely unaffected by *GRH2* and *GRH4*, and selection on these natal hosts showed no visible changes in the fitness of green leafhoppers over 26 generations. However, evaluations on *GRH2/GRH4-*PYL at the end of 20 generations revealed that the nature of colonies selected on *GRH2-*NIL or *GRH4-*NIL had changed. In our no-choice bioassays, nymph biomass and the numbers of eggs laid on *GRH2/GRH4-*PYL were similar between colonies reared on *GRH2-*NIL, *GRH4-*NIL and *GRH2/GRH4-*PYL, indicating an increase in virulence against the pyramided line despite exposure to only one of the genes in that line. In contrast, green leafhoppers from colonies continually exposed to T65 had reduced weight gain and egg laying on *GRH2/GRH4-*PYL. That the colonies reared on *GRH2-*NIL and *GRH4-*NIL could lay eggs on *GRH2/GRH4-*PYL is particularly noteworthy because the pyramided resistance represented a considerable barrier to egg laying. Furthermore, leafhoppers selected on *GRH4-*NIL equally preferred the recurrent parent T65 and *GRH2/GRH4-*PYL, indicating that the leafhoppers had completely adapted to the pyramided line without any exposure to that line. Green leafhoppers selected on *GRH2-*NIL had also adapted to survive, develop and lay eggs on *GRH2/GRH4-*PYL, but did not prefer the line in choice bioassays with T65.

It is difficult to suggest what mechanisms may underlying these observations. It is possible that the insects on *GRH2-*NIL or *GRH4-*NIL experienced difficulties and fitness costs during feeding or egg laying on the monogenic lines, costs that were imperceptible to the experimenters. For example, in a study by Ferrater et al. (2015), despite virulence adaptation in enclosed colonies, planthoppers continued to feed on xylem even after adapting to resistance and attaining similar survival, weight gains and oviposition rates as on susceptible varieties (see also Seo et al., 2010). Such minor fitness costs could have led to a genetic shift in our leafhopper populations toward a predominance of adapted individuals. This assumes that a small proportion of individuals in the original colonies possessed virulence genes (Jing et al., 2014; Kobayashi et al., 2014; Kobayashi, 2016) or were otherwise pre-adapted to the resistance mechanisms associated with *GRH2* and/or *GRH4*. Green leafhoppers may also have responded to volatiles or defence chemicals produced by *GRH2-*NIL or *GRH4-*NIL that predisposed them to survive, develop and lay eggs on *GRH2/GRH4-*PYL, possibly because of induced epigenetic changes associated with host finding and feeding. In a similar study with the brown planthopper, Ferrater and Horgan (2016) demonstrated that planthoppers reared for several generations on the resistant rice variety IR62 (*BPH3/BPH32*) were more virulent than planthoppers reared on susceptible varieties, even when feeding on a novel exposed variety (cv. Triveni). The study indicated that virulent planthoppers had increased their capacity to reduce general rice defences. The study also suggested that virulent planthoppers transmitted unidentified virulence-promoting factors (which could include bacterial symbionts: Wang et al., 2008) that enhanced their feeding ability (Ferrater and Horgan, 2016). A number of studies have implicated bacterial or yeast-like symbionts in virulence adaptation by rice phloem feeders (Lu et al., 2004; Wang et al., 2008; Tang et al., 2010; Ferrater et al., 2013, 2015; Xu et al., 2015; Horgan and Ferrater, 2017); however, the evidence is still largely inconclusive. The green leafhoppers in our study did not carry yeast-like symbionts (Ferrater, unpublished results). However, food-induced changes in the nature of bacterial endosymbionts associated with leafhopper selected on monogenic lines could underlie their improved virulence against the pyramided resistance. Further research is required to test these and other possible mechanisms of virulence adaptation.

### 4.4 Concluding remarks and future research

Our results clearly indicate that pyramided resistance is vulnerable to adaptation and that virulence can result from complicated interactions between herbivores and plant hosts or between defence and virulence mechanisms. In our experiments, the monogenic lines were highly susceptible to green leafhopper attack (possibly enhanced by the high susceptibility of the recurrent parent), which may have facilitated virulence adaptation. Our study did not examine whether the return of green leafhoppers selected on *GRH2*-NIL or *GRH4-*NIL to T65 would result in a loss of virulence against *GRH2/GRH4-*PYL or whether partial virulence could be reversed if selection was relaxed (by returning the leafhoppers to T65 at about the tenth generation of selection). We also did not examine the potential effects of refuge areas with susceptible lines such as T65 on the rates and nature of adaptation to *GRH2-*NIL, *GRH4-*NIL or *GRH2/GRH4-*PYL. It is also difficult to predict whether similar results would have emerged if we had used varieties instead of near-isogenic lines, because quantitative traits associated with the genetic backgrounds of rice varieties can affect herbivore responses to resistance genes (Cohen et al., 1997; Alam and Cohen, 1998; Peñalver Cruz et al., 2011; Horgan, 2018). These aspects of virulence adaptation could be included in future research. Our results indicate that to prolong the durability of pyramided resistance, care should be taken to avoid any long-term exposure to monogenic resistance (including ineffective resistance genes) and to avoid the concurrent deployment of monogenic and pyramided lines (as in the case of *Bt* genes: Zhao et al., 2005). Furthermore, optimal gene combinations could be determined by screening herbivore populations for virulence against key resistance donors and then assessing the proportions of individuals virulent to the different genes or gene combinations (based on individual tests such as honeydew bioassays or swollen abdomens: Myint et al., 2009a, 2009b). We suggest that urgent attention must be placed on understanding the coevolution of crops and herbivores in farmers' fields to help develop informed crop management and deployment strategies that prolong resistance durability.

## Appendix A Supplementary data

Virulence adaptation Virulence adaptation
