# Supplementary material for: Virulence adaptation in a rice leafhopper: Exposure to ineffective genes compromises pyramided resistance
Source: Crop Prot. 2018 Nov;113:40–7. doi: 10.1016/j.cropro.2018.07.010 (PMC6106693; doi:10.1016/j.cropro.2018.07.010)
Supplement: Virulence adaptation [file mmc1.docx]

**Supplementary information 1: Origin of green leafhopper (*Nephottetix virescens*) populations**


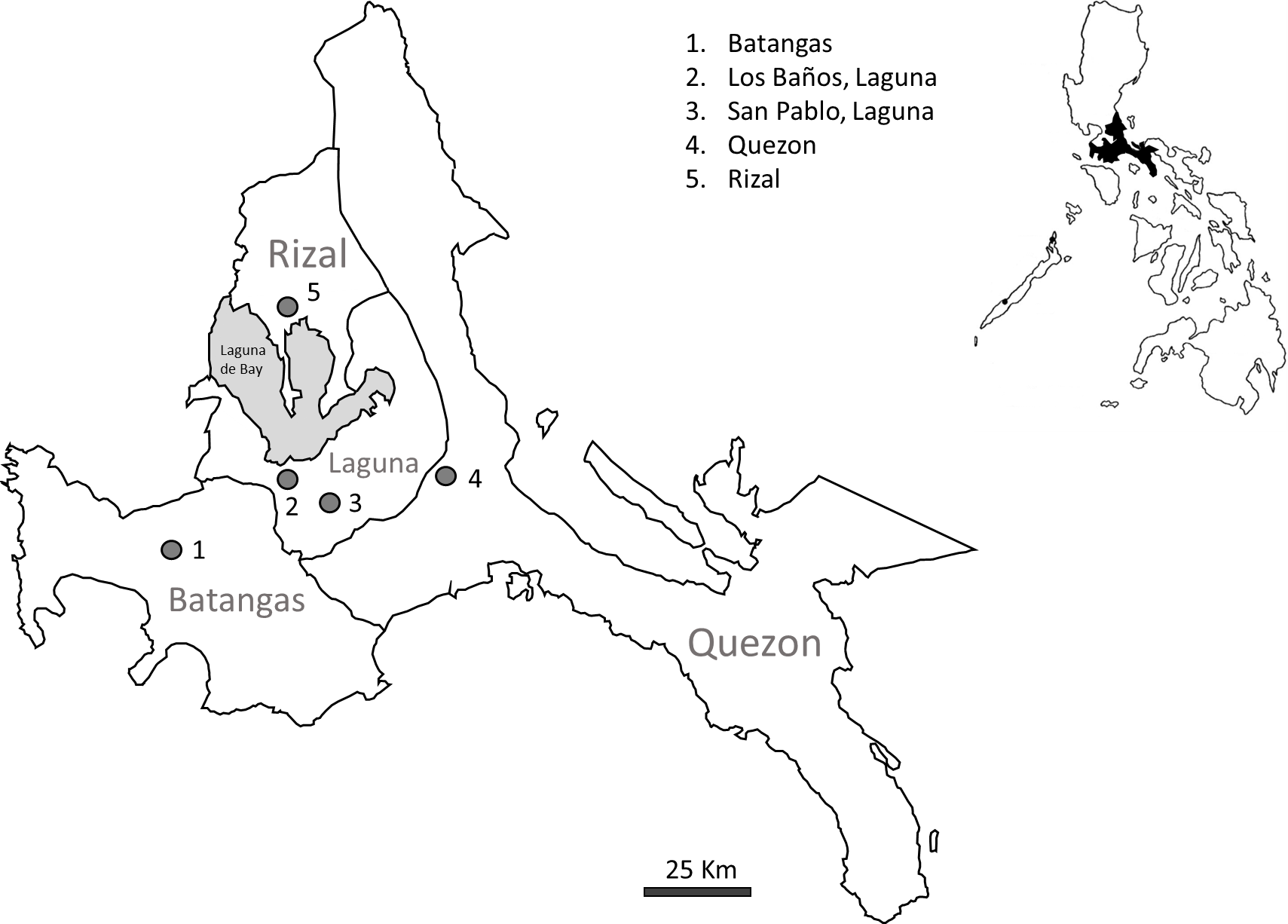


Figure S1: Location origins of five *Nephotettix virescens* populations from southern Luzon (black shading in inset map of Philippines) used to initiate selected colonies. Grey area indicates Laguna de Bay lake.

**Supplementary information 2: baseline study on initial green leafhopper (*Nephottetix virescens*) populations**

**Methods**

The following bioassays were each conducted using five green leafhopper colonies and four rice hosts (T65, *GRH2*-NIL, *GRH4*-NIL, *GRH2/GRH4*-PYL) on five greenhouse (22-37°C, 12D:12N) benches (greenhouse bench = replicated block; N = 5):

Honeydew excretion

Seed of each of the four lines were planted to size-0 pots (5 × 2.5 cm: Height × Radius [H × R]) filled with paddy soil and tended until 20 days after sowing (DAS). The plants received no fertilizer and were not treated with any pesticides. Pots were weeded when necessary and were held in flooded trays such that the soil was saturated throughout the bioassays.

At 20 DAS, plastic chambers (5 ×2.5 cm: H × R) were attached to the lower portions of the plants. The chambers restricted leafhoppers to within 5 cm of the base of the plants and were placed over filter paper that neatly fitted around the shoots. The filter paper had been treated with bromocresol green to indicate the nature of the honeydew as coming from the phloem (basic reaction indicated by blue-rimmed spots) or xylem (acidic reaction indicated by white spots). Newly emerged, unmated, gravid females were added to the cages at 20 DAS. The leafhoppers had been starved for 2 hrs prior to the bioassay. The area of excreted honeydew was measured using Image J software version 1.48 (National Institute of Health, USA). Xylem honeydew as a proportion of total honeydew excretion was used as an indicator of plant resistance.

Adult survival

Adult survival on each rice line was examined by introducing five, unmated adult females to 20 DAS plants. The plants in size-0 pots (5 × 2.5 cm: H × R) were covered with acetate insect cages (45 × 2.5 cm: H × R). The leafhoppers were allowed to feed for 15 days, after which time the number surviving was recorded. The survivors were then collected and dried in a forced draught oven at 60°C for 3 days before being weighted. The feeding plants were also dried and weighed.

Oviposition

Egg-laying was assessed on each rice line by introducing two gravid females to 20 DAS plants prepared as described above (adult survival bioassay). The leafhoppers were allowed to feed and oviposit for 5 days after which the plants were collected and dissected under a light microscope (×10) to count the numbers of egg batches and eggs. The plants were then dried and weighted.

Nymph survival

Nymph survival and development was monitored on plants by introducing ten neonate planthoppers to each rice line. The plants, at 20 DAS, were prepared as above (adult survival bioassay). After 15 days the number of nymphs surviving was recorded. The surviving nymphs were collected and dried before weighing. The developmental stages of the nymphs were also recorded. The proportion of survivors that reached the adult stage was used as an index of development.

Choice adult settling

Settling choice among adult leafhoppers was assessed in a choice bioassay. Plants of each of the four lines were placed inside acetate cages (50 × 50 × 40 cm: Height × Width × Length [H × W × L]; one plant of each line per cage = 4 plants). The plants at 20 DAS in size-0 pots were positioned at the corners of the cages without touching the cage walls or the other plants in the cage. Ten adult females were placed at the centre of each cage. The positions of the adults and conditions of the plants were monitored during six days, ensuring that the plants did not show signs of wilting. After 6 days, the proportions of leafhoppers on each plant were recorded.

Data analysis

Honeydew excretion, nymph survival, nymph biomass, nymph development, adult survival, adult biomass, and the number of eggs laid were analysed using general linear models (GLM) removing the effect of blocks (greenhouse benchs). Plant biomass was initially included in the analyses as a covariate, but was removed where there was no significant effect. The initial models (Type III SS) were as follows: leafhopper response = rice line (L) + population (P) + block + plant weight (covariate) + L×P interaction. Because of non-independence of observations in the choice study, the results per cage (number settling on each line after 6 days) were ranked prior to analysis. Other data transformations are indicated in Table S1. Residuals were plotted after each parametric analysis and found to be normal and homogeneous. Tukey pairwise comparisons were conducted for populations and rice lines after all analyses.

**Results**

All five colonies were capable of feeding and developing on *GRH2-*NIL and *GRH4-*NIL, but had poor survival, low weight gain, slower development and laid fewer eggs on *GRH2/GRH4-*PYL. Three of the populations (Batangas, Los Baños and Rizal) also had significantly delayed nymph development on *GRH4-*NIL compared to nymphs on T65 resulting in a significant P×L interaction, this was not reflected as lower nymph survival; however, the weights of surviving nymphs on *GRH4*-NIL were consistently (though not significant statistically) lower than on T65 or *GRH2-*NIL.

Egg batches produced by leafhoppers from Batangas were larger than those from San Pablo. There was no effect of rice line on average egg batch size (population: F_4,82_ = 3.190, P = 0.017; host plant F_3,82_ = 1.937, P = 0.130, P×L F_12,82_ = 0.898, P = 0.553), and no effect of host plant on the volume of honeydew excreted; however, the proportion of honeydew derived from xylem feeding was higher when leafhoppers fed on *GRH2/GRH4-*PYL (Table S1). Plant biomass (covariate) was only related to nymph survival; lower nymph survival was associated with larger healthier plants.

There were no consistent patterns among the five colonies in their reactions to the different host plants; however, green leafhoppers from Quezon and San Pablo often performed better than the remaining colonies when reared on *GRH2/GRH4-*PYL.

Table S1: Bioassays conducted to examine the fitness of *Nephotettix virescens* before, during and/or after selection on near-isogenic natal hosts

| Bioassay name | Experiments^1^ | Type of bioassay^1^ | infestation stage | infestation density | Plant age^2^ | Duration of bioassay | Data recorded^1^ |
| --- | --- | --- | --- | --- | --- | --- | --- |
| Feeding efficiency | Baseline study on original populations | no-choice | unmated adult females (<1 day) | 1/plant | 20 DAS | 24 hours | Area of xylem-derived honeydew/total honeydew |
| Nymph survival | Baseline study on original populations; Monitoring selection | no-choice | neonates (<1 day) | 10/plant | 20 DAS | 15 days | Number surviving, nymph development stages, final dry weight of nymphs, final dry weight of plant |
| Adult survival | Baseline study on original populations; Monitoring selection | no-choice | unmated adult females (<1 day) | 5/plant | 20 DAS | 15 days | Number surviving, final dry weight of survivors, final dry weight of plant |
| Oviposition | Baseline study on original populations; Monitoring selection | no-choice | mated, gravid females | 2/plant | 20 DAS | 5 days | Number of eggs laid (per surviving female), final dry weight of plant |
| Nymph settling | Feeding efficiency on PYL after 20 generations of selection (4 natal hosts) and six generations of relaxed selection (PYL = natal host) | binary choice (between T65 and PYL) | neonates (<1 day) | 20/cage (2 plants) | 20 DAS | 6 days | Proportion of nymphs on PYL |
| Adult settling | (a) Baseline study on original populations; (b) feeding efficiency on PYL after 20 generations of selection (4 natal hosts) and (c) six generations of relaxed selection (PYL = natal host) | (a) choice between 4 hosts; (b,c) binary choice (between T65 and PYL) | unmated adult females (<1 day) | 16/cage (2 plants) | 20 DAS | 2 days | Proportion of adults on PYL |
| Oviposition preferences | Feeding efficiency on PYL after 20 generations of selection (4 natal hosts) and six generations of relaxed selection (PYL = natal host) | binary choice (between T65 and PYL) | mated, gravid females | 2/cage (2 plants) | 20 DAS | 5 days | Number of eggs laid on each plant, proportion of eggs on PYL |

1: PYL = *GRH2/GRH4-*PYL

2: DAS = Days after sowing

Table S2: Responses (average ± SEM) by five populations of *Nephotettix virescens* from southern Luzon (Philippines) to near-isogenic rice lines with zero (T65 = recurrent parent), one (*GRH2-*NIL, *GRH4-*NIL) and two (*GRH2/GRH4-*PYL) resistance loci

| Exposed host (population origin)^1^ | Honeydew excreted (mm^2^) | xylem (mm^2^)/total honeydew (mm^2^) | Proportion of adults settling^3^ | Percentage of adults surviving (%) | Biomass of adult survivors (d w mg) | Number of eggs laid | Number of egg batches/plant | Percentage of nymphs surviving (%) | Percentage of nymphs developing to adults | Biomass of adult survivors (d w mg) |
| --- | --- | --- | --- | --- | --- | --- | --- | --- | --- | --- |
| (Batangas) |  |  |  |  |  |  |  |  |  |  |
|  | BC | C |  |  | A | BC | B | ABC | B | A |
| T65 | 1.97±0.36 | 0.62±0.13a | 0.43±0.07b | 80.00±5.16b | 4.93±0.52b | 42.00±8.07b | 3.00±0.58ab | 80.00±5.77b | 62.55±8.79c | 5.66±0.42b |
| *GRH2-*NIL | 2.73±0.71 | 0.69±0.10a | 0.24±0.05b | 76.67±6.15b | 3.38±0.30b | 42.50±6.59b | 5.33±0.56b | 83.33±5.58b | 58.89±12.21c | 5.62±0.60b |
| *GRH4-*NIL | 1.46±0.37 | 0.41±0.08a | 0.26±0.07b | 66.67±8.43b | 3.73±0.52b | 52.00±9.55b | 4.67±0.67b | 95.00±3.42b | 15.00±7.19b | 3.69±0.57b |
| *GRH2/GRH4-*PYL | 1.48±0.36 | 1.00±0.00b | 0.07±0.04a | 20.00±5.16a | 0.86±0.21a | 30.17±7.06a | 2.83±0.79a | 1.67±1.67a | 0.00±0.00a | 0.03±0.03a |
| (Los Baños, Laguna) |  |  |  |  |  |  |  |  |  |  |
|  | AB | AB |  |  | AB | AB | AB | A | AB | AB |
| T65 | 1.66±0.34 | 0.30±0.13a | 0.37±0.05b | 83.33±6.15b | 5.27±0.80b | 22.17±6.00b | 2.17±0.48ab | 70.00±5.16b | 31.61±7.11c | 5.14±0.45b |
| *GRH2-*NIL | 1.32±0.14 | 0.23±0.10a | 0.24±0.04b | 80.00±8.94b | 5.13±0.67b | 33.17±15.74b | 3.00±1.18b | 75.00±6.71b | 34.52±16.94c | 5.89±0.58b |
| *GRH4-*NIL | 1.38±0.21 | 0.29±0.09a | 0.32±0.02b | 90.00±6.83b | 5.51±0.62b | 39.17±7.78b | 5.00±0.86b | 70.00±6.83b | 21.51±6.56b | 4.70±0.35b |
| *GRH2/GRH4-*PYL | 0.69±0.32 | 1.00±0.00b | 0.07±0.02a | 26.67±6.67a | 0.91±0.19a | 6.00±3.87a | 0.67±0.42a | 8.33±3.07a | 0.00±0.00a | 0.11±0.04a |
| (Quezon) |  |  |  |  |  |  |  |  |  |  |
|  | A | A |  |  | AB | D | C | C | B | AB |
| T65 | 1.61±0.48 | 0.20±0.08a | 0.34±0.06b | 90.00±6.83b | 4.92±0.52b | 81.83±10.09b | 7.17±1.28ab | 91.67±4.77b | 32.12±5.42b | 6.28±0.47b |
| *GRH2-*NIL | 1.00±0.12 | 0.17±0.08a | 0.29±0.08b | 83.33±6.15b | 3.75±0.52b | 78.83±8.21b | 8.50±1.09b | 90.00±4.47b | 45.42±8.07b | 6.51±0.53b |
| *GRH4-*NIL | 1.38±0.29 | 0.30±0.09a | 0.33±0.05b | 70.00±13.42b | 4.26±0.92b | 80.67±17.90b | 7.83±1.60b | 95.00±3.42b | 30.28±9.07b | 5.96±0.38b |
| *GRH2/GRH4-*PYL | 0.56±0.27 | 0.85±0.13b | 0.04±0.02a | 33.33±6.67a | 1.34±0.32a | 47.00±12.72a | 4.67±0.80a | 30.00±13.66a | 0.00±0.00a | 0.45±0.20a |
| (Rizal) |  |  |  |  |  |  |  |  |  |  |
|  | AB | AB |  |  | AB | C | B | AB | B | AB |
| T65 | 1.54±0.35 | 0.22±0.10a | 0.29±0.06b | 83.33±3.33b | 4.99±0.24b | 39.00±10.76b | 3.67±0.71ab | 86.67±4.22b | 32.98±3.73c | 5.93±0.31b |
| *GRH2-*NIL | 1.15±0.28 | 0.33±0.06a | 0.35±0.05b | 73.33±4.22b | 4.52±0.23b | 47.67±6.10b | 5.00±0.58b | 86.67±2.11b | 37.73±7.63c | 6.04±0.29b |
| *GRH4-*NIL | 1.41±0.18 | 0.42±0.08a | 0.33±0.04b | 80.00±5.16b | 4.81±0.17b | 59.33±8.37b | 5.33±0.33b | 70.00±14.61b | 18.05±4.23b | 4.37±0.90b |
| *GRH2/GRH4-*PYL | 0.87±0.25 | 0.99±0.01b | 0.02±0.02a | 20.00±5.16a | 1.02±0.27a | 30.17±7.65a | 3.00±0.58a | 3.33±2.11a | 0.00±0.00a | 0.04±0.03a |
| (San Pablo, Laguna) |  |  |  |  |  |  |  |  |  |  |
|  | C | BC |  |  | B | A | A | BC | A | B |
| T65 | 1.96±0.57 | 0.44±0.11 | 0.36±0.05b | 80.00±7.30b | 4.86±0.60b | 17.67±4.88b | 2.50±0.85ab | 85.00±5.63b | 9.26±6.03b | 5.61±0.40b |
| *GRH2-*NIL | 2.11±0.48 | 0.43±0.10 | 0.29±0.02b | 80.00±7.30b | 4.91±0.77b | 23.33±8.88b | 3.50±1.45b | 88.33±3.07b | 31.06±10.85c | 6.02±0.48b |
| *GRH4-*NIL | 1.64±0.28 | 0.45±0.08 | 0.26±0.04b | 90.00±4.47b | 6.24±0.53b | 14.33±8.29b | 1.83±1.08b | 86.67±6.67b | 6.53±3.05b | 4.75±0.58b |
| *GRH2/GRH4-*PYL | 2.80±0.45 | 0.99±0.01 | 0.09±0.03a | 33.33±9.89a | 1.82±0.52a | 5.00±2.73a | 0.83±0.40a | 25.00±11.47a | 0.00±0.00a | 0.43±0.26a |
| Transformation | None | None | Rank | Arcsine | Log+1 | None | None | Arcsine | Rank | Log+1 |
| F-Population (P) | 6.664*** | 7.120*** |  | 1.524 | 2.602* | 22.848*** | 19.575*** | 5.865*** | 5.887*** | 3.679** |
| F-Rice line (L) | 1.838 | 58.841*** | 50.210*** | 73.192*** | 83.928*** | 7.561*** | 10.592*** | 57.004*** | 52.589*** | 365.220*** |
| Covariate (plant weight)^2^ |  |  |  |  |  |  |  | 14.064*** |  |  |
| F-P×L | 1.749 | 1.242 |  | 0.81 | 0.870 | 0.489 | 0.787 | 1.177 | 2.340** | 0.789 |
| Error DF | 95 | 95 | 119 | 95 | 95 | 95 | 95 | 94 | 95 | 95 |

1: Uppercase letters indicated homogenous population groups (Tukey P < 0.05) and lowercase letters indicate homogenous rice-line groups (Tukey P ≤ 0.05)

2: The covariate ‘plant biomass’ was initially included in all models, but removed where non-significant.

3: Adult settling was examined in a choice bioassay and was not compared across populations. Data were ranked because of non-independence of observations

**Supplementary information 3: stability of green leafhopper (*Nephottetix virescens*) virulence against *GRH2/GRH4*-PYL**

**Methods**

After 20 generations, 100 pairs of adult (male and female) green leafhoppers were collected from each *GRH2/GRH4-*PYL-selected colony (N=5 colonies) and placed in rearing cages (50 × 50 × 40 cm: H × L × W) with T65. The colonies were maintained on T65 for six generations. After six generations, a series of bioassays was conducted with (a) *GRH2/GRH4-*PYL-virulent colonies that were continuously maintained on *GRH2/GRH4-*PYL, (b) *GRH2/GRH4-*PYL-virulent colonies that were returned to T65 for six generations, and (c) colonies that had been continuously reared on T65 for 26 generations (total = 15 colonies). Bioassays included nymph survival, adult survival, and egg laying tests as described for the baseline study (see also Table S1).

A series of binary-choice bioassays were also conducted with each of the 15 colonies to examine preferences between the recurrent parent T65 and the *GRH2/GRH4*-PYLs (Table S1). In the choice bioassays, T65 and *GRH2/GRH4-*PYL plants at 20 DAS (one of each plant) were placed inside acetate cages (50 × 50 × 40 cm: H × L × W) without touching each other or the cage walls. Plants were assessed daily throughout the duration of each bioassay to ensure that they did not show signs of wilting.

Nymph settling on the plants was assessed by placing 20 neonate green leafhoppers at the centre of the cages and recording their positions (occurrence on plants) after 6 days. Adult settling on the plants was assessed by placing 16 adult green leafhoppers at the centres of each cage and monitoring their positions after 2 days. Adult preferences for egg-laying were assessed by placing two gravid females at the centre of each cage. After 5 days the adults were removed and the plants collected. The plants were dissected under a light microscope to count the eggs.

**Results**

Colonies selected on *GRH2/GRH4-PYL* did not lose virulence to the pyramided line after they were returned to T65 and reared for six generations. The original GRH2/GRH4-selected colonies and the colonies returned to T65 performed similarly in each choice (Figure S2) and no-choice (Figure S3) bioassay without any apparent reduction in virulence.

Adult survival (F_2,12_ = 4.285, P = 0.039: Figure S2A) and biomass (F_2,12_ = 10.214, P = 0.003: Figure S2B) was higher for leafhoppers returned to T65 for 6 generations than for leafhoppers continuously reared for 26 generations on T65 (trends for biomass were similar to survival and are not presented). The biomass of *GRH2/GRH4-*PYL plants at the end of the bioassays was higher after feeding by green leafhoppers from the T65 colonies (F_2,12_ = 7.540, P = 0.008: Figure S2C). Females from the T65 colonies laid fewer eggs on *GRH2/GRH4-*PYL plants compared to those selected on the pyramided line or returned to T65 for 6 generations (F_2,12_ = 17.555, P < 0.001: Figure S2D). Nymph survival was high for all colonies (77-82%: F_2,12_ = 0.936, P = 0.421); however, nymphs from T65 colonies gained significantly less weight during the bioassay (F_2,12_ = 6.305, P = 0.013: Figure S2E) and fewer individuals developed to adults (F_2,12_ = 38.000, P < 0.001: Figure S2F). The history of the colonies had no effect on *GRH2/GRH4-*PYL biomass during the nymph survival bioassay (F_2,12_ = 0.230, P = 0.798).

During the choice bioassays, fewer nymphs from colonies reared on T65 settled on *GRH2/GRH4-*PYL compared to nymphs from selected colonies (F_2,12_ = 24.706, P < 0.001: Figure S3A). At the end of the bioassay, *GRH2/GRH4-*PYL plants attacked by selected colonies that were returned to T65 for 6 generations, had less biomass (were more damaged) than plants attacked by T65 reared colonies (F_2,12_ = 5.609, P = 0.019: Figure S3B). Green leafhoppers from the selected colonies (26 generations on *GRH2/GRH4-*PYL and 20 generations on *GRH2/GRH4-*PYL + 6 generations on T65) laid similar numbers of eggs on the PYL and T65 in the choice experiments; leafhoppers continually reared on T65 deposited few of their eggs on the PYL (F_2,12_ = 15.319, P < 0.001: Figure S3C). There was no significant difference in the numbers of eggs laid during the bioassay by any of the colonies (F_2,12_ = 2.000, P = 0.178: Figure S3D).

Figure S2: Results from bioassays with green leafhopper colonies that were continuously reared on *GRH2/GRH4-*PYL during 26 generation (GRH2/4), that were reared on *GRH2/GRH4-*PYL for 20 generations and then on T65 for six generations (T65), and that were continually reared on T65 for 26 generations (T65*). Only *GRH2/GRH4-*PYL was used in the bioassays. Graphs indicate (A) the proportion of adults surviving on *GRH2/GRH4-*PYL after 5 days, (B) the dry weight of adult survivors on *GRH2/GRH4-*PYLs after 5 days, (C) the dry weigh of the *GRH2/GRH4-*PYL at the end of the adult survival bioassay, (D) the number of eggs laid (E) the biomass of nymphs at the end of the survival bioassay, and (F) the proportion of nymphs developing to adults after 10 days on *GRH2/GRH4-*PYL. Lowercase letters indicate homogenous groups (Tukey tests, P < 0.05); standard errors are indicated (N = 5 colonies). White bars indicate that colonies had no previous history of exposure to GRH2/GRH4-PYL prior to the bioassays.

Figure S3: Results from choice settling and egg-laying bioassays with green leafhopper colonies that were continuously reared on *GRH2/GRH4-*PYL during 26 generation (GRH2/4), that were reared on *GRH2/GRH4-*PYL for 20 generations and then on T65 for six generations (T65), and that were continually reared on T65 for 26 generations (T65*). Graphs indicate (A) the proportion of adults settling on *GRH2/GRH4-*PYLs in binary choice experiments with T65, (B) the final biomass of *GRH2/GRH4-*PYL plants in binary choice bioassays with T65, (C) the proportion of eggs laid on *GRH2/GRH4-*PYLs and (D) the total numbers of eggs laid in the experiments. Lowercase letters indicate homogenous groups (Tukey tests, P < 0.05); standard errors are indicated (N = 5 colonies). White bars indicate that colonies had no previous history of exposure to *GRH2/GRH4-*PYL.
